# Extra-Gastrointestinal Stromal Tumor (EGIST) in the Pelvis Mimicking Retroperitoneal Sarcoma

**DOI:** 10.1055/s-0042-1757335

**Published:** 2022-09-26

**Authors:** Etienne El-Helou, Linda Chamma, Houssam Bashir Mazraani, Delivrance Sebaaly, Omar Georges Chamma, Jessica Naccour, Marwan M. Haddad, Dani Lichaa, Houssam Alam

**Affiliations:** 1 Department of General Surgery, Lebanese University, Faculty of Medical Sciences, Beirut, Lebanon; 2 Department of Anatomic Pathology, Lebanese University, Faculty of Medical Sciences, Beirut, Lebanon; 3 Department of Internal Medicine, Lebanese University, Faculty of Medical Sciences, Beirut, Lebanon; 4 Department of Emergency Medicine, Lebanese University, Faculty of Medical Sciences, Beirut, Lebanon; 5 Department of Radiology, Mount Lebanon Hospital, Beirut, Lebanon; 6 Department of General Surgery, Centre Hospitalier Universitaire Geitaoui, Beirut, Lebanon

**Keywords:** extra-gastrointestinal, stromal tumors, GIST, retroperitoneal sarcoma, surgery, case report

## Abstract

Extra-gastrointestinal stromal tumors (EGISTs) are rare mesenchymal tumors accounting for less than 1% of total gastrointestinal tumors. They tend to be aggressive and have a poor prognosis. Unfortunately, there is a lack of data or controversial data due to its scarcity. Therefore, we report a case of pelvic EGIST misdiagnosed as retroperitoneal sarcoma. We opted for surgical management followed by adjuvant oral chemotherapy with imatinib.

Gastrointestinal stromal tumors (GISTs) are among the most common mesenchymal tumors‎ 1 of the gastrointestinal (GI) tract; they account for approximately 3% of all GI tumors, with up to 70% found in the stomach, 25% in the small intestine, 5% in the colon and rectum, and 5% in the esophagus. 2 The cell that gives rise to these tumors is a pluripotent mesenchymal stem cell that has been automated to differentiate into interstitial Cajal cells‎, 2 which are found in intestinal wall,‎ 3 and regulate its peristalsis. 2

Extra-gastrointestinal stromal tumors (EGISTs), as the term implies, are GISTs found outside the GI tract, 2 3 representing for less than 5% of total GISTs, 3 and less than 1% of total GI tumors‎. 2 EGISTs are most commonly found in the omentum, mesentery, retroperitoneum, pelvic region, abdominal wall, liver, gallbladder, pancreas, bladder, seminal vesicle, vagina prostate, 3 and scrotum‎. 2 The majority of cases involve elderly patients, ranging from 45.8 to 59 years as mean age and are larger than GISTs at the time of diagnosis‎. 1

EGISTs share histopathological and molecular characteristics with GISTs‎, 2 but unlike GISTs, some research has shown that EGISTs are derived from Cajal cells or pluripotent stem cells found outside the GI tract. 3 Histopathologically, they are divided into spindle, epithelioid, and mixed types‎. 1

There is a lack of data or controversial data because it is a rare pathology and little studied as an independent entity‎. 1 The exact incidence of EGISTs and their tumor behavior is unknown. 2

Here, we present a case of EGIST found incidentally in an elderly patient, who was surgically treated and followed by adjuvant oral chemotherapy.

## Case Description

We present a case of 75-year-old male patient, with an unremarkable medical history, presented to outpatients clinics in another institution for 2 months history of urinary tract symptoms including dysuria, incontinence, and small volume of urine when he voids. A urinary tract infection was suspected and the patient was, therefore, treated with a course of antibiotics.

Unfortunately, the antibiotic therapy did not alleviate the patient's symptoms, so he presented to our clinics. A palpable soft mass at the lateral wall of the rectum was found on physical examination. A colonoscopy was performed, and no abnormal findings were found.

**Fig. 1:**
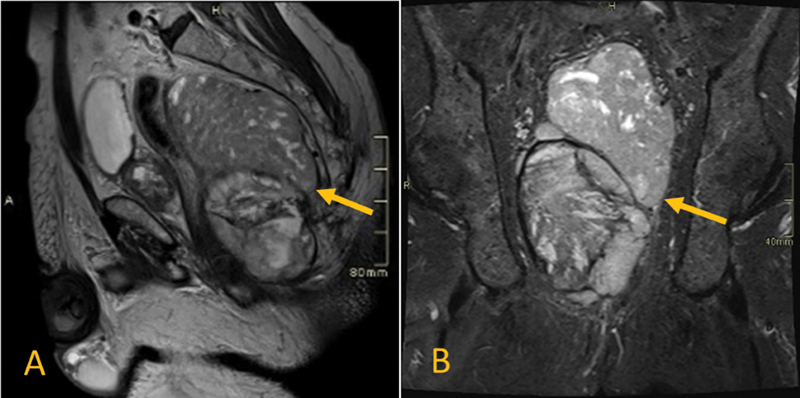
The pelvic lesion shows heterogeneous signal on T2 ( **A** ) and short tau inversion recovery ( **B** ) weighted images (all arrows point to tumor).

Subsequently, the pelvic magnetic resonance imaging revealed a 14.5 × 8.6 × 8.1cm pelvic heterogeneous exophytic mass that shows heterogeneous T2, mixed hyper- and hypo-T1 signal intensities (denoting hemorrhagic components), heterogeneously enhanced and demonstrating areas of restricted diffusion on diffusion-weighted imaging ( Figs. 1 2 3 ).

**Fig. 2:**
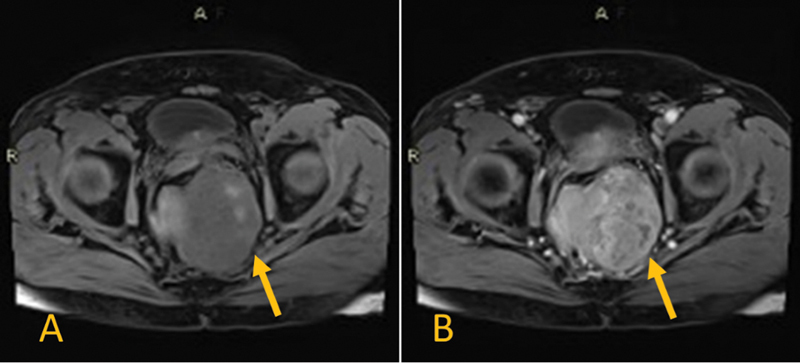
The pelvic lesion shows hyposignal T1 ( **A** ) and enhancement after injection of gadolinium ( **B** ) (all arrows point to tumor).

**Fig. 3:**
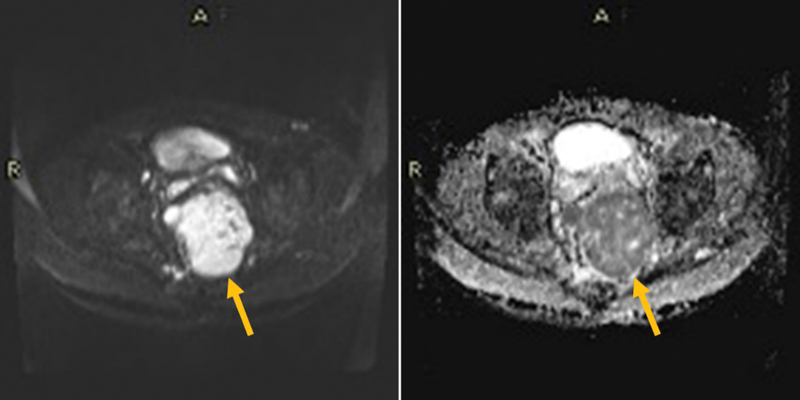
The pelvic lesion shows restriction on diffusion and apparent diffusion coefficient sequences.

**Fig. 4:**
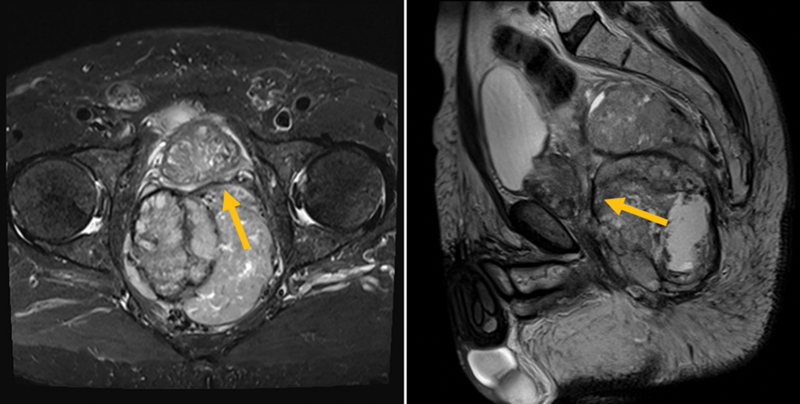
The lesion shows mass effect on the rectum that is compressed to the left with invasion of the mesorectal fascia. It also indents the posterior border of the prostate.

It shows mass effect on the rectum that is compressed to the left with invasion of the mesorectal fascia and the pelvic side walls. It also indents the posterior border of the prostate ( Fig. 4 ). Subcentimetric pelvic retroperitoneal lymph nodes are seen, the largest seen in the right internal iliac chain measuring 6 mm.

The mass biopsy was inconclusive with the suspicion of having a retroperitoneal sarcoma as one of the differential diagnoses; hence, we opted for surgical resection.

On the day of the surgery, a bilateral double J stent was put in place. Laparoscopically, exploration and dissection through the pelvic cavity revealed the presence of the mass that was in firm contact with the posterior part of the bladder, the right ureter, and the mesorectum. Invasion of these structures could not be ruled out by laparoscopy; therefore, conversion to open surgery was performed, where total tumor excision was performed along with partial cystectomy and partial excision of the mesorectum. A protective loop ileostomy was performed for fear of having iatrogenic or ischemic rectal lesions after the operation.

The patient tolerated the surgery well and was subsequently discharged home without major complications.

**Fig. 5:**
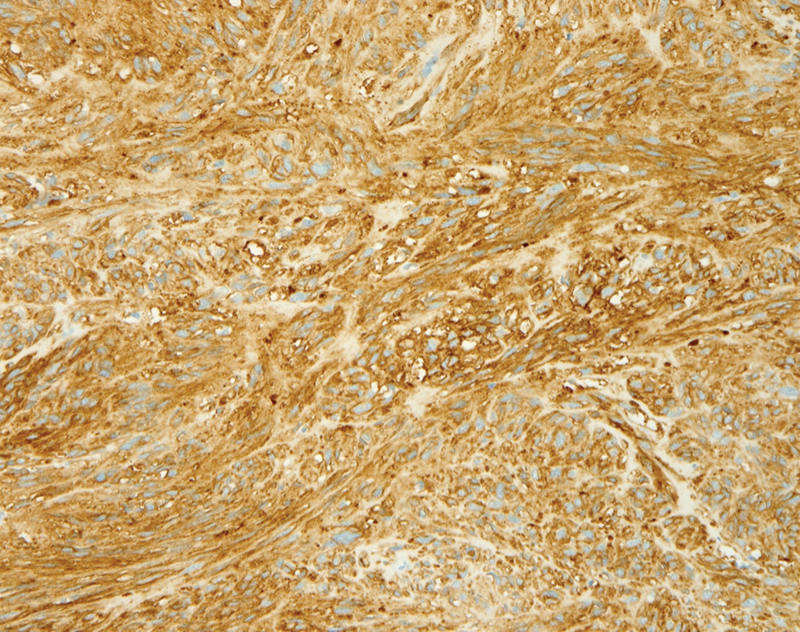
Immunohistochemistry showing staining for discovered on gastrointestinal stromal tumor 1 protein. Original magnification x200.

**Fig. 6:**
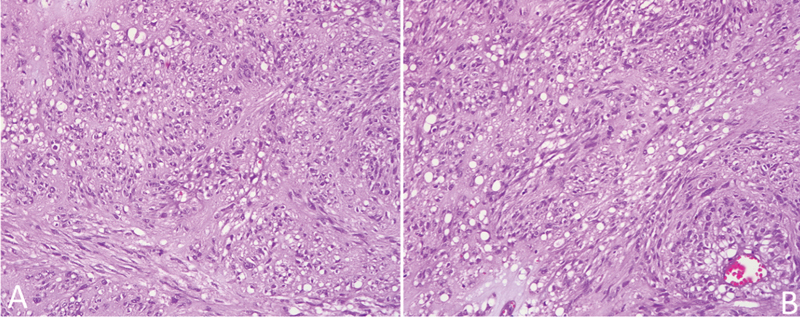
( **A** ) Hematoxylin and eosin stain (HE) showing spindle cells. ( **B** ) HE showing epithelioid cells. Original magnification x200.

**Fig. 7:**
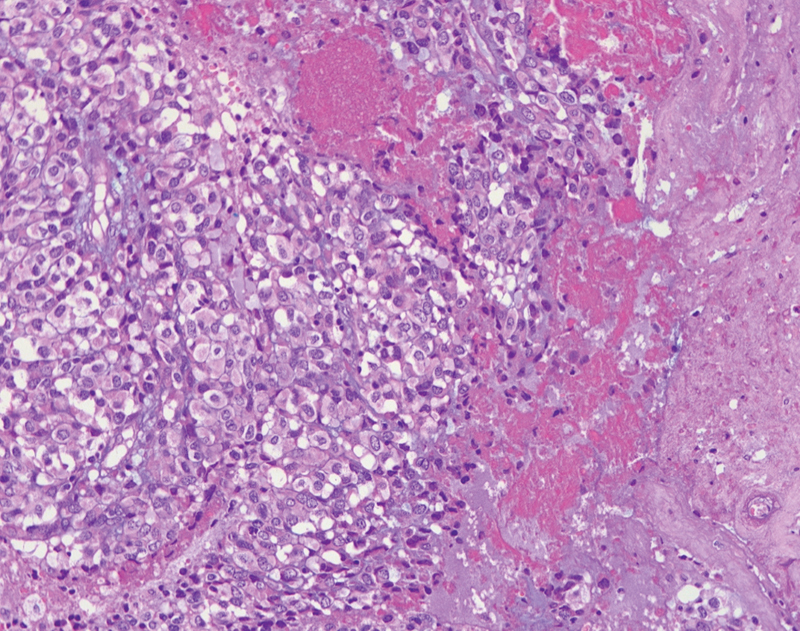
Hematoxylin and eosinx200.2 showing areas of necrosis with epithelioid cells.

The pathology report and the immunochemistry study confirmed the diagnosis of GIST, by the diffuse expression of CD117 as well as discovered on GIST-1 (DOG1; Fig. 5 ) and h-Caldesmon. In addition, since both spindle and epithelioid cells were distinguished, this tumor was classified as a mixed cell type ( Fig. 6A and B ). Grossly, areas of necrosis and hemorrhages among the epithelioid cells were described ( Fig. 7 ). The mitotic rate was 3.

By combining histology and morphology, this tumor as described corresponded to the criterion of malignant EGIST.

The patient started adjuvant chemotherapy with imatinib. In follow-up, he showed a good response, no reported side effects, and no signs of recurrence.

## Discussion

EGISTs are rare tumors found in the nondigestive tract‎, 4 which affect more commonly women and the elderly patients‎. 1 They are more commonly seen in larger sizes 1 and advanced stage due to the delay in the diagnosis‎. 4

They are identified according to the diagnostic characteristics of GIST (Anapath: abbreviation of Anatomopathological examination, immunohistochemistry, and molecular genetic analysis). There are studies that have suggested that EGIST is a secondary lesion or metastatic deposit of GIST, but others have shown that it is a special entity with its own characteristics. 4

EGISTs have no specific symptoms or radiological features; the most common presentation is abdominal discomfort or pain‎, 2 and these vary depending on the tumor size and location. 1 They are discovered incidentally during investigations for abdominal pain, abdominal mass, or during a laparotomy. 3

Patient history, physical examination, appropriate blood work, and imaging studies, as well as surgical evaluation, may be included in the EGIST workup. Endoscopy and endoscopic ultrasound can be helpful in some cases‎. 2 An enhanced computed tomography scan usually reveals some distinguishing features, such as a large mass with peripheral enhancement of unknown origin shifting adjacent structures and possible abdominal lymphadenopathy nodes. 3

Imaging alone does not distinguish EGIST from other abdominal pathologies‎ 3 ; to confirm the diagnosis, a percutaneous transperitoneal biopsy or an endoscopic transmural biopsy is required, 2 and immunohistochemistry studies reveal that the cells are positive for immunological markers such as CD117, DOG1, CD34, B-cell lymphoma 2, desmin, smooth muscle actin, 3 and S100. 1 The combination of CD117 and DOG1 improves significantly diagnosis accuracy. 3

The differential diagnosis tends to be broad and includes leiomyosarcoma, liposarcoma, fibrosarcoma,‎ 2 3 solitary fibrous tumor, 2 or lymphoma. 3

The treatment of EGIST is similar to that of GIST, which consists of radical surgical en bloc resection with negative margins. 3 4 The efficacy of combination with imatinib as neoadjuvant or adjuvant treatment remains controversial; however, it is recommended for the treatment of unresectable tumors, with dose modification according to the disease progression. 3

EGISTs are more aggressive than GISTs, but have a poor prognosis, 1 with 5-year survival rate reaching 48.9%. 2 They have a low mitotic index of 5/50 high-power fields and less, and tend to metastasize in up to 30% of cases. 1 Mitosis, necrosis, and large tumor size are considered high-risk factors for a poorer prognosis. 4 The recurrence rate has been reported up to 23%. 1

Failure to explore clinicopathological differences between EGIST and GIST may have resulted in inappropriate treatment approaches, or it may have contributed to EGIST's poor prognosis‎. 4 Long-term follow-up studies are needed to recognize the behavior of this rare neoplastic disease. 2

## Conclusion

EGISTs are rare mesenchymal tumors with a poor prognosis. They are discovered incidentally and confirmed by the combination of immunohistochemistry CD117 and DOG1 detection. The clinical presentation can be tricky, so we need to be rigorous and keep this differential diagnosis in mind. We encourage researchers to further explore this pathology to obtain clear guidelines on treatment and outcomes.
